# Synchronous Occurrence of Papillary Thyroid Carcinoma and Medullary Carcinoma in the Setting of Hashimoto’s Thyroiditis and Multi Nodular Goiter 

**DOI:** 10.30699/IJP.2021.527288.2606

**Published:** 2020-10-20

**Authors:** Fatemeh SamieeRad, Ali Emami

**Affiliations:** 1 *Department of Pathology, Faculty of Medical School, Qazvin University of Medical Sciences, Qazvin, Iran*; 2 *Student Research* *Committee, Qazvin University of Medical Sciences,* *Qazvin, Iran*

**Keywords:** Hashimoto’s thyroiditis, Medullary carcinoma, Multinodular goiter, Papillary thyroid carcinoma, Synchronous neoplasm

## Abstract

Coexistence of follicular epithelial and bilateral parafollicular cells derivative of carcinomas in the setting of Hashimoto’s thyroiditis and multinodular goiter is a very rare event. Of course, all benign and malignant thyroid lesions are more prevalent in iodine deficient areas. It seems that the context for identifying the pathways influencing thyroid carcinogenesis especially coincidence form has not yet been fully understood and needs further investigation. Here, we present a case with the synchronous occurrence of papillary thyroid carcinoma and medullary thyroid carcinoma in the setting of Hashimoto’s thyroiditis and multinodular goiter. A 54-year-old woman complained of a painless mass in the anterior region of the neck. The physical examination of the patient revealed multiple nodules in her thyroid gland. In ultrasound findings, she presented with thyroid enlargement associated with multiple isoechoic and hypoechoic nodules in both lobes. Thyroid fine needle aspiration results suggested a diagnosis of medullary thyroid carcinoma in the setting of Hashimoto’s thyroiditis and multinodular goiter . The frozen sections, permanent sampling, and IHC examination showed the coexistence of papillary thyroid carcinoma with bilateral medullary thyroid carcinoma in the setting of Hashimoto's disease and multinodular goiter . Studies debated about the risk factors of these pathologies including the same environmental issues or mutations in genomes and they emphasized surgeons should be aware of these lesions for diagnosis and interventional treatments. Following up the Hashimoto’s thyroiditis and multinodular goiter is required for detection ofoccult malignancies, and hence the proper management and treatment should be performed.

## Introduction

The most prevalent endocrine malignancy is thyroid cancers (TC) (1), and the incidence of this malignancy has extremely increased over the last decades due to new effective diagnostic approaches and the detection of small nodules (2). 

Papillary thyroid carcinoma (PTC) consists 85% of thyroid cancers (3). The source of this cancer is follicular thyroid cells and PTC is known as the seventh most common cancer among women and the second-fastest-growing tumor among men (1, 4). The risk factors of PTC has been documented as exposure to ionizing radiation, female gender, smoking, obesity, dietary iodine excess, alcohol, dietary nitrates, diabetes, and genetic factors (5). PTC is related to exclusive mutations, including BRAF V600E, RAS, and chromosomal rearrangements leading to the expression of tyrosine kinase receptors such as RET, NTRK, and ALK (2). Lymph node metastasis in the neck is over 75% of the large tumors and 50% of the small tumors. PTC has a high recovery rate and patients with PTC diagnosis have long-term prognoses (6).

Medullary thyroid carcinoma (MTC) is the third most common thyroid carcinoma and parafollicular cells are its sources. It is much rarer than PTC (1-3% of all thyroid cancers). Most MTC is sporadic, and 25% of MTC are associated with other endocrinopathies, including multiple endocrine neoplasia type 2 (MEN2) and familial medullary thyroid carcinoma (FMTC) (7). The clinical aggressiveness of MTC relies on *RET* proto-oncogene (8). The recovery rate in MTC is much lower than the other thyroid cancers. This carcinoma is the most aggressive type of thyroid cancer, and the rate of relapse and mortality is much higher than that in PTC (6).

Hashimoto's thyroiditis (HT) is the autoimmune inflammatory disorder of the thyroid gland leading to the demolition of the parenchyma with decreased T3 and T4 levels and elevation of thyroid-stimulating hormone (TSH). It is the most common autoimmune pathology of this gland. HT is one of the most important reasons of hypothyroidism in endocrinology (8).

The prevalence of HT is 0.3-1.5 cases out of 1000 cases per year. HT has two variants: the nodular form and the diffuse form. The characteristic features of nodular form are fibrosis, sclerosis, and calcifications and are particularly associated with PTC (9). The BRAF mutation V600E was controversial in association with cancer and HT (9).

The earlier studies have revealed that PTC is common among patients with autoimmune lymphocytic thyroiditis, while others have debated these findings (10). The correlation is explained by the dysregulation of follicular epithelial following inflammatory injury; however, the molecular pathogenesis is unclear (11). The correlation of HT and well-differentiated thyroid cancer may demonstrate a less aggressive clinical presentation and better prognosis (12). 

The concurrence of MTC and PTC is rare and occurs in less than 0.5% of all thyroid cancers. The management, treatment, and follow-up should be done accordingly. There is no difference between the biological behavior of MTC in association with PTC cases with solitary MTC cases (6).

The coexistence of follicular epithelial cells and bilateral parafollicular cells derivative of carcinomas in the setting of HT and multinodular goiter is a very rare event. Of course, all benign and malignant thyroid lesions are more prevalent in iodine-deficient areas. It seems that the context for identifying the pathways influencing thyroid carcinogenesis, especially coincidence form has not yet been fully understood and needs further investigation. Data accumulation of more cases will indeed provide further experiences.

In this case, we described the concurrence of MTC and PTC in an Iranian woman with synchronous HT and multinodular goiter (MNG) and associated literature review. There has been no report of the synchronous occurrence of these four thyroid pathologies together to the best of our knowledge.

## Case Presentation

The patient was a 54-year-old woman who complained of a painless mass in the anterior region of the neck, which had grown progressively since about nine months ago. She presented with constipation in accompanying symptoms. She had a history of hypothyroidism, and a history of irregular daily use of levothyroxine . Her familial history for endocrine disease was also negative.

 The physical examination of the patient revealed multiple nodules in her thyroid gland, but her cervical lymphadenopathy was negative. In laboratory findings, hypothyroidism and anemia were also detected.

In ultrasound findings, she presented with thyroid enlargement associated with multiple isoechoic nodules in the thyroid gland. Additionally, there were hypoechoic nodules in the right lobe, M: 3.5*3.5*2 cm and two another in the left lobe, M: 4.5*3.5*2.5 cm, and M: 1.3*1.3*1 cm. Background constituted multiple ill-defined isoechoic nodules. No cervical lymph node was detected.

Thyroid scintigraphy (99 m –Tc MIBI) reported multiple warm nodules in both lobes of the thyroid gland. Furthermore, there was a cold nodule in the right lobe, M: 3*3*2 cm and two another in the left lobe, M: 4*3*2.5 cm and 1 cm in diameter. Background showed thyroiditis.

Thyroid fine needle aspiration results showed hypercellular smear, including clusters and singly dispersed pleomorphic plasmacytoid tumoral cells. Individual tumoral cells were characterized by eccentric nuclei, some of them nucleated, salt and pepper chromatin, inconspicuous nucleoli and abundant amphophilic cytoplasm with obvious red granules. Moreover, there were many Hurthle cells with high nuclear-cytoplasmic ratio, some bi-nucleated forms, moderate to abundant cytoplasm admixed with many lymphocytes in colloid and bloody background. Therefore, diagnosis of MTC in the setting of HT and MNG was considered.

The patient underwent total thyroidectomy. Sampling was done for frozen section examination. During surgery, cervical lymphadenopathy was not observed. The macroscopic examination of the right and left lobes showed two well-defined unencapsulated gray-tan masses, M: 3*3*2 cm and M: 4.5*3*2.5 cm in the upper pole. Cut surfaces showed non-homogenous gray-tan-yellow, firm tissue with hemorrhage.

Additionally, there was an encapsulated creamy solid nodule, 1 cm in diameter, in the left lobe. Multiple variable-sized colloid nodules were also seen. The cut surface of other areas resembled lymph nodes with tannish color.

**Fig. 1. A, B:**
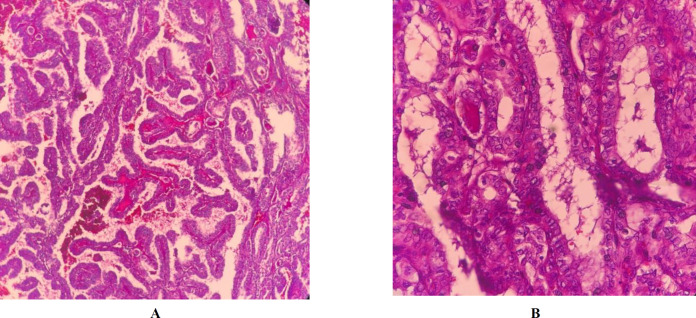
Papillary thyroid carcinoma, (X40 and 400, H&E)

**Fig. 2. A, B:**
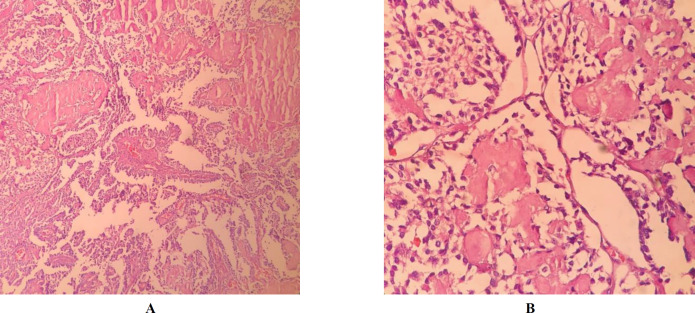
Medullary carcinoma with amyloid depositions (X40 and 400, H&E)

**Fig. 3:**
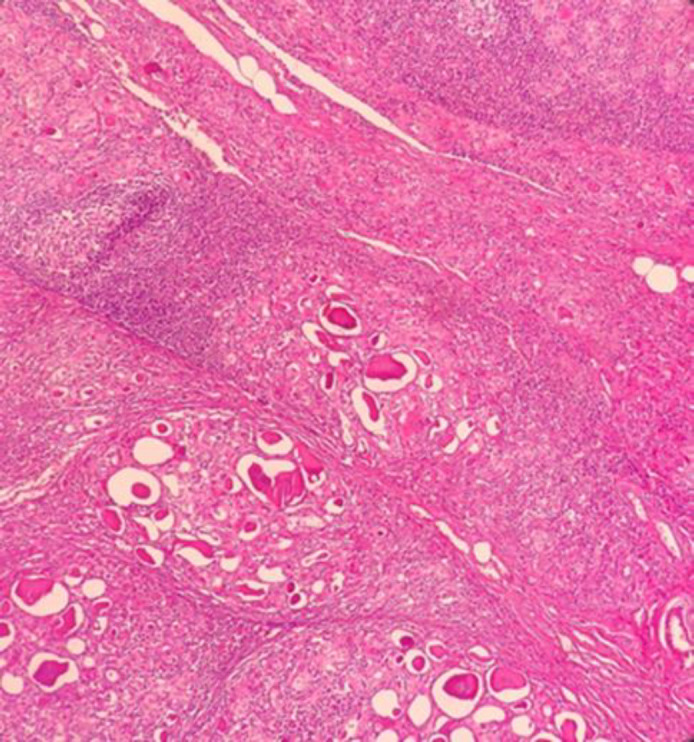
Hashimoto's thyroiditis with nodule formation, lymphoid follicle and Hurthle cells (X40, H&E)

Frozen section examination and permanent sampling slides showed the coexistence of PTC (papillary structure populated by tumoral cells with high N/C ratio, ground glass nuclei with molding, groove and pseudo nuclear inclusion) (Figure 1 A, B) with bilateral MTC (Figure 2 A, B) in the setting of Hashimoto's disease (Figure 3) and MNG . 

**Fig. 4:**
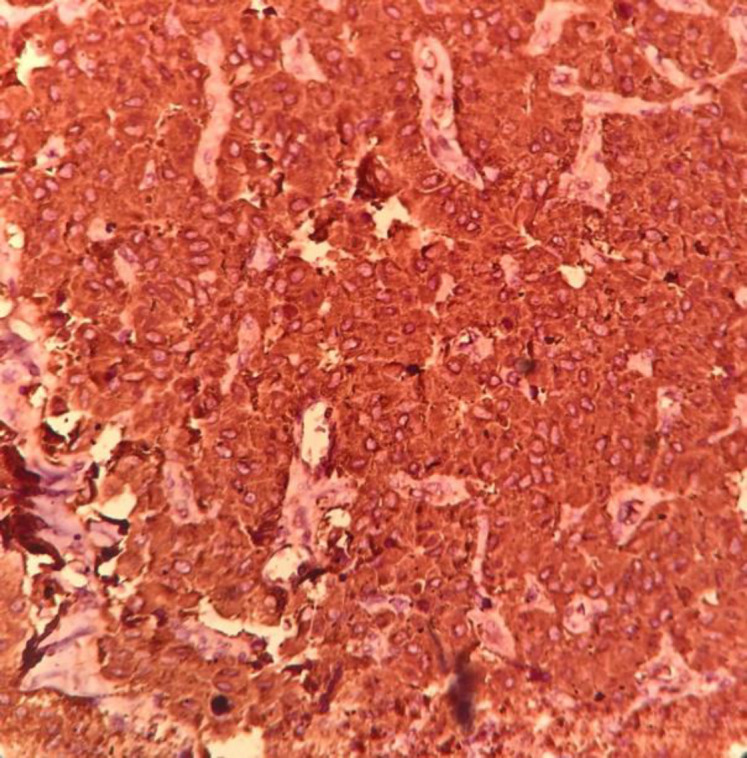
Calcitonin expression in medullary carcinoma (IHC, 200).

**Fig 5:**
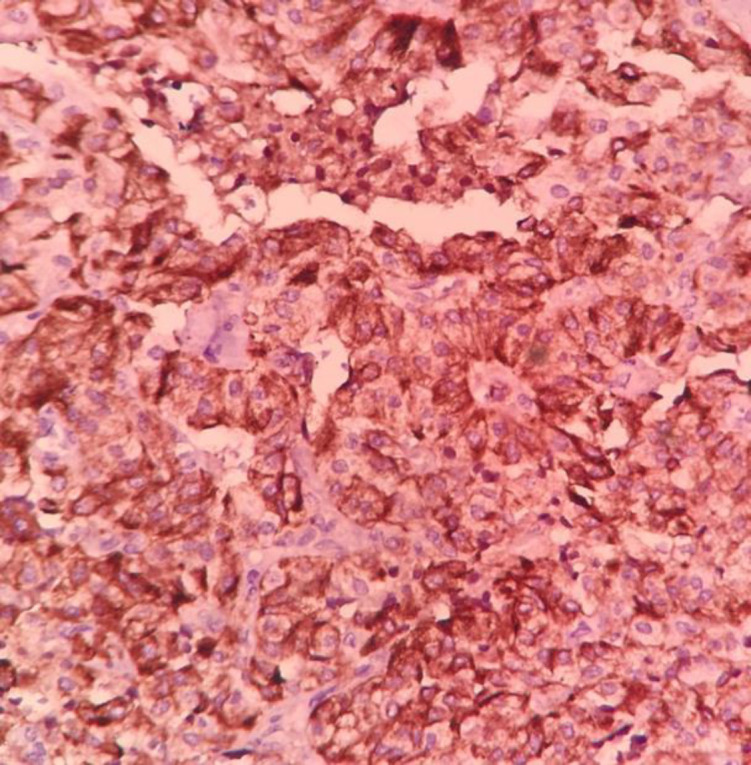
CK19 expression in papillary thyroid carcinoma (IHC, 200)

Immunohistochemical examination showed positive cellular expression for calcitonin (Figure 4) in medullary carcinoma and CK19 (Figure 5) in PTC components. Screening for RET gene mutations was negative. 

After surgery, she was fine and her vital signs were stable. She was referred to the oncology ward, where radioactive iodine therapy was administered. A whole-body I-131 Scan was done during the following up, which showed no evidence of regional lymph node or recurrence. The patient was free of disease in the following 17 months. The participant informed consent was obtained before enrollment in this study.

## Discussion

The histopathologic findings of our case are consistent with the synchronous occurrence of PTC, MTC, HT, and MNG. 

Besides thyroid cancers, the synchronous occurrence of these cancers with HT is common (13). The presence of thyroid cancers and MNG is rare. One study determined the frequency of thyroid cancer in MNG in patients for whom thyroidectomy was performed. The authors reported the frequencies of PTC and undifferentiated thyroid cancer were 6% and 2%, respectively, among all patients (14). The possibility of concurrence of HT, PTC, and MTC depends on some gene rearrangements (5, 6, 9, 13). Besides these synchronous occurrences, HT has been introduced as playing a protective role against PTC progression (15). Researchers have found biomarkers that are a transformer of HT to PTC. These biomarkers include p63 expression, PI3K/Akt expression, RET/PTC rearrangements, and BRAF mutation (5). In addition to genetics, immune-mediated pathways linking PTC and HT were discovered. Cellular mediators such as CD3+, CD4+, and Th17 produced by immune cells might have caused a malignant transformation in the thyroid gland (16). This link between one malignancy and one autoimmune disease is a controversial question.

The epidemiology of simultaneous MTC and PTC was mentioned in a study by Appetecchia *et al*. In that study, 109 patients were cured for both carcinomas at the last follow-up and 6 patients died from MTC. The median time to progression was 123 months (95% confidence interval (CI): 89.3-156.7 months). They reported that when MTC and PTC synchronously occurred, the preference should be given to the management and treatment of PTC since it associates with the severe impact on prognosis (2).

Environmental risk factors can increase the risk of thyroid cancers. Cupisti *et al.* studied a 52-year-old patient with synchronous MTC, PTC, FTC, and recurrent goiter. In his pathohistological examination, the biopsy showed a 5 cm FTC, a 0.3 cm PTC in the right lobe, and a 1.5 cm MTC in the left lobe of the thyroid gland. In this case, the *RET* germline mutation was ruled out. Their patient was a core maker in a foundry and they reported the main risk factor was long-term exposure to carcinogens such as hydrazine. This exposure played an important role in his condition (17). Unlike the mentioned case, our patient had no history of contact with any carcinogens. Our case had no history of recurrent goiter before reference to the doctor. Like the case in above study, our patient had nodules in both lobes of her thyroid. 

In the histopathological examination of our case, Hurthle cells along with numerous lymphocytes were reported. These findings showed the inflammation such as thyroiditis at the base of thyroid cancers. This inflammation could progress to a malignancy. 

In their study, Molnar *et al.* examined the clinicopathological features of the TC and HT co-occurrence. They reported PTC could be noticed as an outcome for HT by special characteristics other than BRAF and NRAS mutations, and also PTC can be diagnosed earlier to the prevention of metastatic dissemination (18). In our case, the RET germline mutation was negative. On the other hand, Rio *et al. *reported a higher presence of thyroiditis in malignant thyroid diseases rather than benign ones. They found PTC was related to thyroiditis more than other thyroid pathologies; however, the correlation between thyroiditis and higher prevalence of thyroid cancers remained veiled (12). 

## Conclusion

The synchronous concurrence of papillary thyroid carcinoma (PTC), medullary thyroid carcinoma (MTC), Hashimoto’s thyroiditis (HT), and multinodular goiter (MNG) is a rare pathology of the thyroid gland. Studies debated about the risk factors of these pathologies, including the same environmental factors or mutations in genomes and those emphasized on awareness of surgeons of such lesions for making diagnosis and interventional treatments. Following up the HT and MNG is required for detection occult malignancies, and hence the proper management and treatment should be performed.

## Authors' Contributions

The first author (Fatemeh Samiee-Rad,) contributed to the study conception and design. Material preparation, data collection and case presentation were performed by (Fatemeh Samiee-Rad). The first draft of the manus-cript was written by [Fatemeh Samiee-Rad and Ali Emami], and both authors commented on previous ver-sions of the manuscript. All authors read and approved the final manuscript. 

## Conflict of Interest

The authors declare that there are no conflicts of interest regarding the publication of this paper. All applicable international, national and/or institutional guidelines followed. Before students were enrolled in this study, participant consent was obtained.
